# Preoperative CT-Based Deep Learning Model for Predicting Risk Stratification in Patients With Gastrointestinal Stromal Tumors

**DOI:** 10.3389/fonc.2021.750875

**Published:** 2021-09-17

**Authors:** Bing Kang, Xianshun Yuan, Hexiang Wang, Songnan Qin, Xuelin Song, Xinxin Yu, Shuai Zhang, Cong Sun, Qing Zhou, Ying Wei, Feng Shi, Shifeng Yang, Ximing Wang

**Affiliations:** ^1^ Cheeloo College of Medicine, School of Medicine, Shandong University, Jinan, China; ^2^ Department of Radiology, Shandong Provincial Hospital Affiliated to Shandong University, Jinan, China; ^3^ Department of Radiology, The Affiliated Hospital of Qingdao University, Qingdao, China; ^4^ Department of Radiology, Hospital of Traditional Chinese Medicine of Liaocheng City, Liaocheng, China; ^5^ School of Medicine, Shandong First Medical University, Jinan, China; ^6^ Department of Research and Development, Shanghai United Imaging Intelligence Co., Ltd., Shanghai, China

**Keywords:** gastrointestinal stromal tumors, risk assessment, deep learning, tomography, X-ray computed, prediction model

## Abstract

**Objective:**

To develop and evaluate a deep learning model (DLM) for predicting the risk stratification of gastrointestinal stromal tumors (GISTs).

**Methods:**

Preoperative contrast-enhanced CT images of 733 patients with GISTs were retrospectively obtained from two centers between January 2011 and June 2020. The datasets were split into training (n = 241), testing (n = 104), and external validation cohorts (n = 388). A DLM for predicting the risk stratification of GISTs was developed using a convolutional neural network and evaluated in the testing and external validation cohorts. The performance of the DLM was compared with that of radiomics model by using the area under the receiver operating characteristic curves (AUROCs) and the Obuchowski index. The attention area of the DLM was visualized as a heatmap by gradient-weighted class activation mapping.

**Results:**

In the testing cohort, the DLM had AUROCs of 0.90 (95% confidence interval [CI]: 0.84, 0.96), 0.80 (95% CI: 0.72, 0.88), and 0.89 (95% CI: 0.83, 0.95) for low-malignant, intermediate-malignant, and high-malignant GISTs, respectively. In the external validation cohort, the AUROCs of the DLM were 0.87 (95% CI: 0.83, 0.91), 0.64 (95% CI: 0.60, 0.68), and 0.85 (95% CI: 0.81, 0.89) for low-malignant, intermediate-malignant, and high-malignant GISTs, respectively. The DLM (Obuchowski index: training, 0.84; external validation, 0.79) outperformed the radiomics model (Obuchowski index: training, 0.77; external validation, 0.77) for predicting risk stratification of GISTs. The relevant subregions were successfully highlighted with attention heatmap on the CT images for further clinical review.

**Conclusion:**

The DLM showed good performance for predicting the risk stratification of GISTs using CT images and achieved better performance than that of radiomics model.

## Introduction

Gastrointestinal stromal tumors (GISTs) are mesenchymal neoplasms that mostly originate from the gastrointestinal tract with variable malignant potential, which ranges from small lesions with a benign behavior to aggressive sarcomas (1) and account for 1% to 2% of gastrointestinal neoplasms (2). The prevalence of GISTs is about 130 cases per million population (1, 3, 4). Evaluation of malignancy risk of GISTs is mainly based on tumor size, location, and mitotic count through postoperative specimens. These factors are combined in the National Institutes of Health (NIH) risk category criteria (5), which stratify GISTs into four risk categories: very low, low, intermediate, and high-risk tumors. An accurate preoperative categorization of risk classification can provide valuable information for evaluating the adequacy of surgical resection and the need for adjuvant treatment (6, 7).

Contrast-enhanced CT is widely recognized as the main imaging method for the diagnosis, characterization, and evaluation of curative effect in GIST patients (8, 9). In recent years, multiple researches have evaluated the predictive CT imaging features of the risk stratification of GISTs (10–13). However, these subjective assessments are likely affected by the individual experience and heterogeneous definition of imaging features (14–17). Radiomics, which transforms medical images into mineable high-dimensional data, allows to quantify lesion heterogeneity, which cannot be evaluated by the naked eye (18, 19). Several studies have shown that radiomics based on CT scan was of certain value for the prediction of malignancy in GISTs (20–24).

Nevertheless, the radiomics approach depends heavily on handcrafted feature engineering, which is vulnerable to human biases and may result in a high superfluity of information (25). Deep learning, as one of the powerful algorithms of representation learning, has recently been widely applied in the field of diagnostic imaging and prediction owing to their advantages of being fast, accurate, and reproducible (26, 27). Theoretically, the risk stratification of GISTs by deep learning may yield a great diagnostic approach. However, to the best of our knowledge, this is the first-ever study that investigates whether deep learning could be used as a tool to predict risk stratification in GISTs.

Moreover, most of the existing studies assessing the risk stratification of GISTs are based on single-center data, which introduce bias to a model and limit its applicability. In this multicenter study, we further investigate if a quantitative CT-based deep learning approach can objectively predict the risk stratification of GISTs, by developing and validating a deep learning-based model on a large collection of patient data from two different institutions.

## Materials and Methods

### Characteristics of Patients

This two-center retrospective study was approved by the institutional review board of both Shandong Provincial Hospital and The Affiliated Hospital of Qingdao University. Patient informed consent was waived for this retrospective analysis.

**Figure 1:**
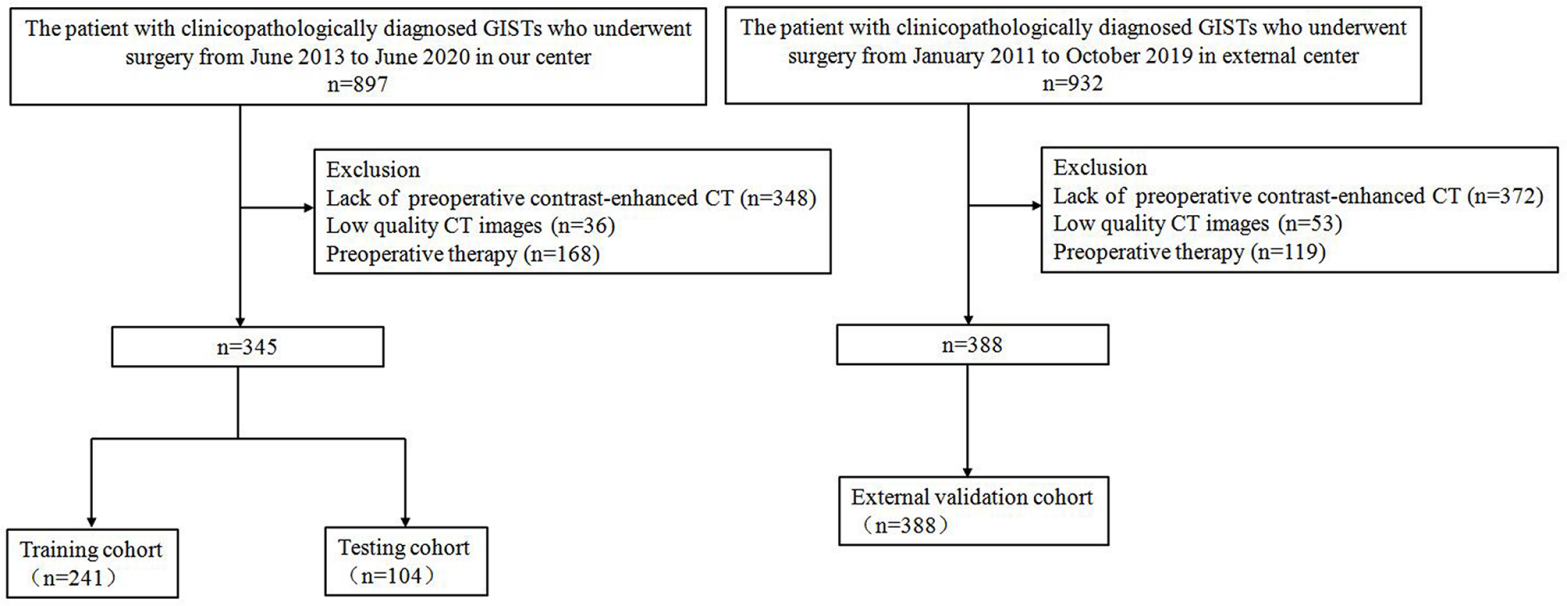
Flow chart of patient inclusion and exclusion.

The inclusion and exclusion criteria of the patients are presented in **Supplementary Material 1.1**. From January 2011 to June 2020, a total of 733 patients (352 men; mean age, 59.8 ± 10.1 years) with GISTs were enrolled in this retrospective study. The study population flow chart is illustrated in **Figure 1**. Demographic and clinicopathologic characteristics, including age, gender, tumor location, tumor size, and mitotic count, were derived from medical records. The modified NIH criteria were used to stratify the malignant potential of GISTs (5), as a verification of our model (**Supplementary Table 1**). According to risk categories, the patients in this study were divided into the low-malignant (very low and low risk), intermediate-malignant (intermediate risk), and high-malignant (high risk) potential groups.

### CT Image Acquisition and Tumor Segmentation

All 733 patients underwent abdominal contrast-enhanced CT examination covering the whole tumor. CT image acquisition and retrieval procedure are described in **Supplementary Material 1.2**. The regions of interest (ROIs) containing the entire tumor were manually drawn on each CT image slice in arterial, venous, and delayed phases with ITK-SNAP software (Version 3.6.0, www.itksnap.org). The ROIs were drawn by one radiologist and confirmed by another (BK and XW, with 6 and 20 years of experience, respectively, in abdominal imaging); both were aware of the diagnosis of GISTs but blinded to the NIH risk stratification. Besides, we randomly selected 30 patients with three-phase CT image segmentation, and we compared the inter-reader agreement for image segmentation by Dice similarity coefficient (DSC).

### Image Preprocessing

Data augmentation has been proven to help prevent network overfitting and memorization of the exact details of the training images. In our study, the following augmentations are applied: rotation, scaling, and flipping (**Supplementary Material 1.3**).

Due to the imbalance of class number used in this study, the information of the rare class may be ignored because it might be underrepresented during training. To handle this problem, a strategy of oversampling the rare classes was applied.

In our dataset, the tumor size ranges from 10 to 240 mm (**Supplementary Figure 2**), which made it challenging to crop the ROIs containing the complete tumor from the original images using a suitable patch size. Therefore, we proposed an adaptive strategy according to the tumor size to preprocess the samples (**Supplementary Material 1.3**), which could ensure that the patches can contain the complete tumor region for big tumors and not overscale for small tumors. Next, each input patch was first normalized by Z-score standardization method, where the voxel intensity was subtracted by 40 and then divided by 250 and subsequently clipped to an intensity range of [−1, 1].

### Development of the Deep Learning Model

**Figure 2:**
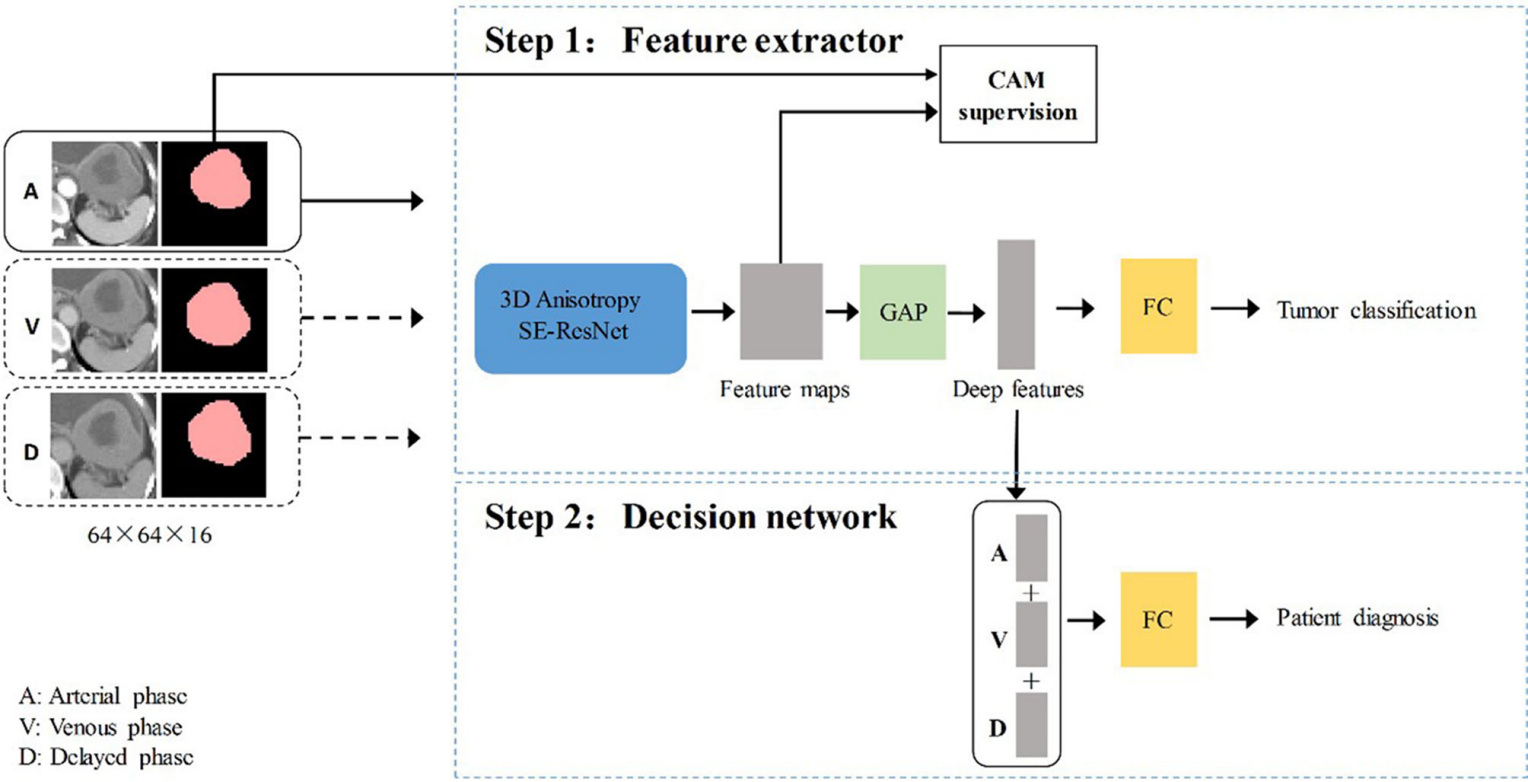
The overall gastrointestinal stromal tumors risk stratification framework. CAM, class activation mapping; A, arterial phase; V, venous phase; D, delayed phase; FC, fully connected layer; GAP, global average pooling layer.

The training of the deep learning model (DLM) involved two steps: 1) tumor feature extraction and tumor classification; and 2) multi-sequence-based feature fusion and patient diagnosis. A detailed framework is described in **Figure 2**. Residual neural network (ResNet) was applied to train the image data and to build our neural network model (**Supplementary Material 1.4**). To provide more insight for model decisions, an attention heatmap of the GISTs was generated by gradient-weighted class activation mapping (CAM) and then superimposed on the original CT images so that the location of the actual tumor and the region highlighted by the model could be compared.

#### Deep Learning Network for Extracting Risk Stratification-Related Features

**Figure 3:**
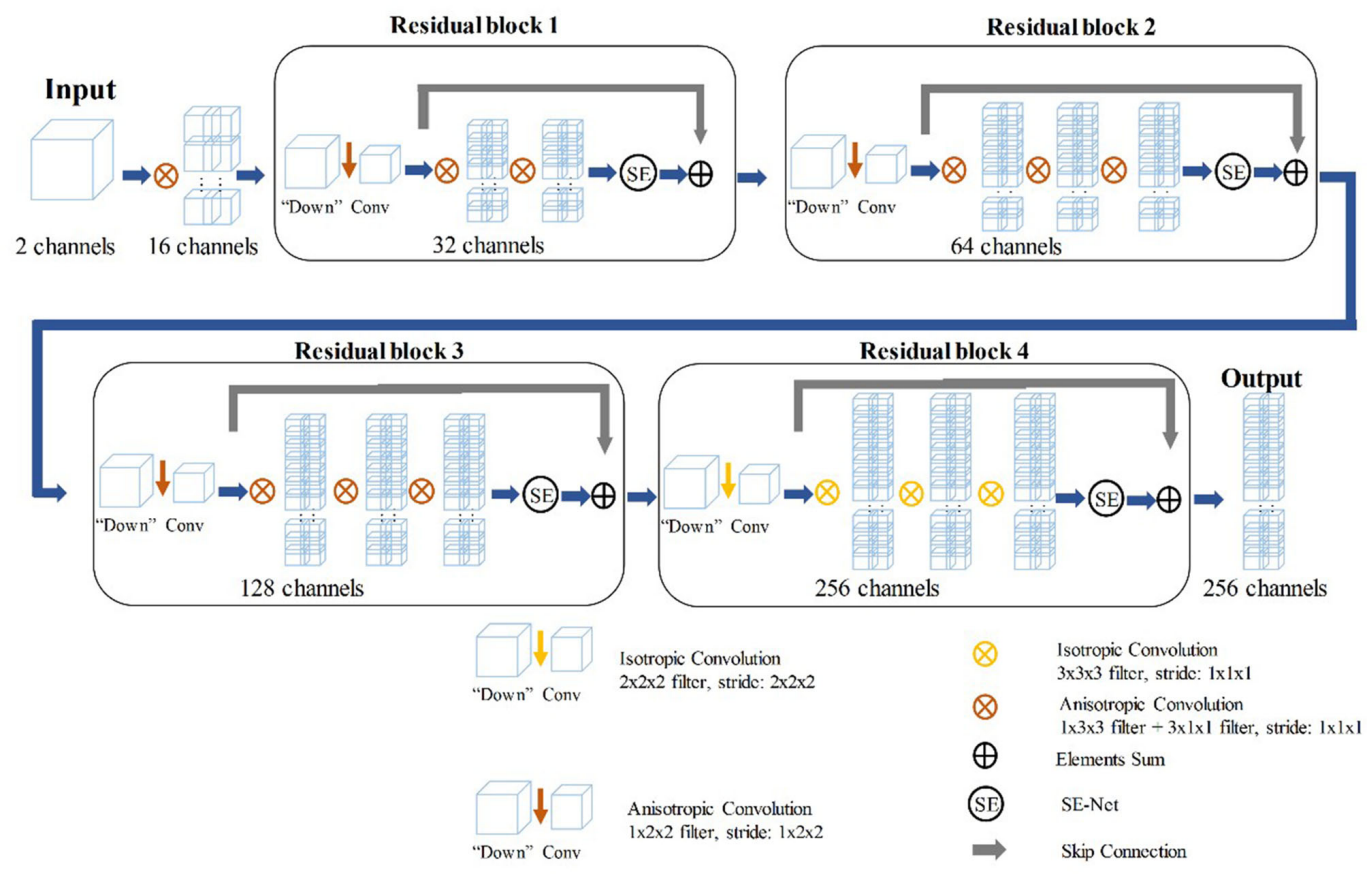
The structure of 3D SE-Residual Network.

In the training stage, we propose to treat the arterial, venous, and delayed phase images as independent samples to optimize the network in the tumor level. We extracted deep features from the three-phase images of each patient by using 3D SE-Residual Network (28) to learn the GIST risk stratification-related features (**Figure 3**). In this scheme, a total of 723 tumor samples (141 × 3 for low-malignant, 43 × 3 for intermediate-malignant, and 57 × 3 for high-malignant) were used as training data in the feature extractor network.

#### The Decision Network for Patient Diagnosis

The three-phase deep learning features extracted by the network are concatenated as a column feature vector, which is then addressed to the classification network for training.

#### Training Details

The network architecture is implemented in PyTorch and trained using NVIDIA Apex for less memory consumption and faster computation. In our experiments, all the models are trained from scratch, in four NVIDIA TITAN RTX graphics processing units, and the inference time for one sample is approximately 4.6 s in one NVIDIA TITAN RTX GPU.

#### Ablation Study

To evaluate the impact of hyperparameters, such as the different loss function combinations on the model classification performance, we adopted a strategy to gradually add the loss function to assess the different loss functions’ contribution to the model.

### Development of the Radiomics Model

In the radiomics model construction, a total of 2,600 quantitative radiomics features were extracted from each tumor in each phase using Pyradiomics package in Python software (29). Details of the radiomics features are shown in **Supplementary Material 1.5**. The three-phase extracted features were subsequently combined for model construction. Considering the relatively large number of features, the least absolute shrinkage and selection operator (LASSO) regression model was performed to select the most valuable features in the training cohort. The support vector machine (SVM) classifier was then used to develop the radiomics model with a five-fold cross-validation strategy in the training set. For the SVM classifier, a radial basis function (RBF) kernel is used, and the hyperparameters were automatically optimized for the best performance in the training set by using Bayesian optimization method, instead of randomly predefining hyperparameters as in conventional classifiers.

### Statistical Analysis

Statistical analyses were conducted with R Studio (version 1.3.959) and Python (version 3.7) with p-value of less than 0.05 considered as statistical significance. To evaluate the performances of DLM and radiomics model, we adopted five different metrics: areas under the receiver operating characteristic curves (AUROCs), accuracy (ACC), sensitivity (SEN), specificity (SPE), and F1 score (F1). AUROCs with 95% confidence interval (CI) were calculated. Moreover, the Obuchowski index was used to evaluate the significant level of difference in diagnostic accuracy of DLM and radiomics model, which is a non-parametric estimation method of the AUROCs adapted for ordinal or nominal scale.

## Results

### Patient Characteristics

A total of 733 GIST patients were split into three independent cohorts: the training, testing, and external validation cohorts. Three hundred forty-five patients (152 men; mean age, 59.1 ± 10.3 years) from Shandong Provincial Hospital diagnosed between June 2013 to June 2020 were randomly assigned to either the training cohort [241 patients (99 men; mean age, 59.2 ± 10.5 years)] and or the testing cohort [104 patients (53 men; mean age, 58.9 ± 9.8 years)] in a 7:3 ratio, using a stratified random split in patient level. The external validation cohort consisted of 388 patients (200 men; mean age, 60.4 ± 9.9 years) diagnosed with GIST from The Affiliated Hospital of Qingdao University between January 2011 and October 2019. In the training cohort, 141 (58.5%) were low-malignant GISTs, 43 (17.8%) were intermediate-malignant GISTs, and 57 (23.7%) were high-malignant GISTs. In the testing cohort, 61 (58.7%) were low-malignant GISTs, 18 (17.3%) were intermediate-malignant GISTs, and 25 (24.0%) were high-malignant GISTs. In the external validation cohort, 137 (35.3%) were low-malignant GISTs, 67 (17.3%) were intermediate-malignant GISTs, and 184 (47.4%) were high-malignant GISTs. Patient characteristics in the training, testing, and external validation cohorts are presented in **Table 1**.

**Table 1:** Characteristics of patients.

Characteristic	Training cohort	Testing cohort	External validation cohort
No. of patients	241	104	388
Age* (years)	59.2 ± 10.5 (19–82)	58.9 ± 9.8 (28–80)	60.4 ± 9.9 (21–83)
Gender			
Male	99 (41.1)	53 (51.0)	200 (51.5)
Female	142 (58.9)	51 (49.0)	188 (48.5)
Site			
Gastric	192 (79.7)	86 (82.7)	236 (60.8)
Non-gastric	49 (20.3)	18 (17.3)	152 (39.2)
Size (cm)			
<2	44 (18.3)	20 (19.2)	19 (4.9)
2.1–5.0	122 (50.6)	53 (51.0)	148 (38.1)
5.1–10.0	59 (24.5)	25 (24.0)	138 (35.6)
>10	16 (6.6)	6 (5.8)	83 (21.4)
Mitotic count			
≤5/50	193 (80.1)	82 (78.8)	247 (63.7)
6–10	27 (11.2)	12 (11.5)	73 (18.8)
>10	21 (8.7)	10 (9.6)	68 (17.5)

Unless otherwise specified, data in parentheses are percentages.

*Numbers in parentheses are the range.

### Diagnostic Performance of the Deep Learning Model

**Figure 4:**
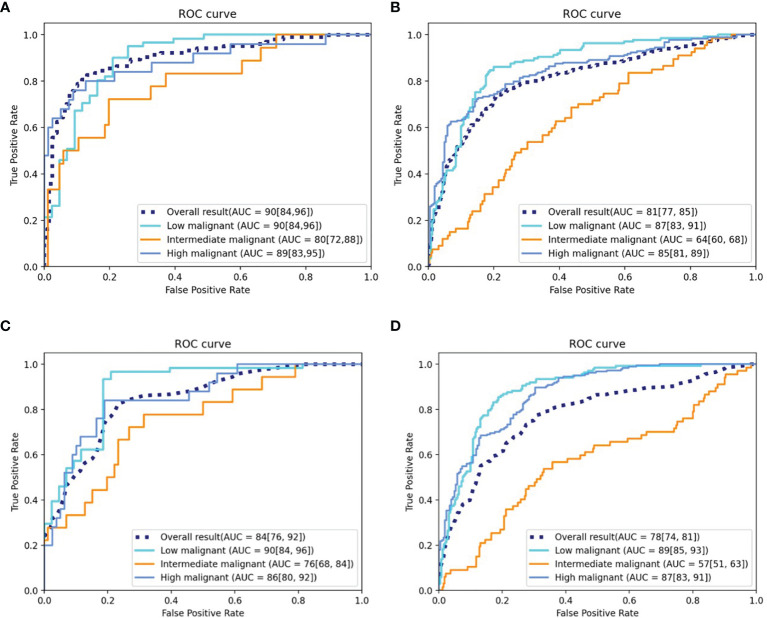
ROC curves of the DLM and radiomics model. **(A)** ROC curve of the DLM for the testing cohort. **(B)** ROC curve of the DLM for the independent external validation cohort. **(C)** ROC curve of the radiomics model for the testing cohort. **(D)** ROC curve of the radiomics model for the independent external validation cohort. ROC, receiver operating characteristic; AUC, area under the curve; DLM, deep learning model.

The DLM achieved good performance in assessing risk stratification of GISTs with the use of CT images, with the overall AUROCs of 0.90 (95% CI: 0.84, 0.96) in the testing cohort and 0.81 (95% CI: 0.77, 0.85) in the external validation cohort. The ROCs are shown in **Figures 4A, B**.

**Figure 5:**
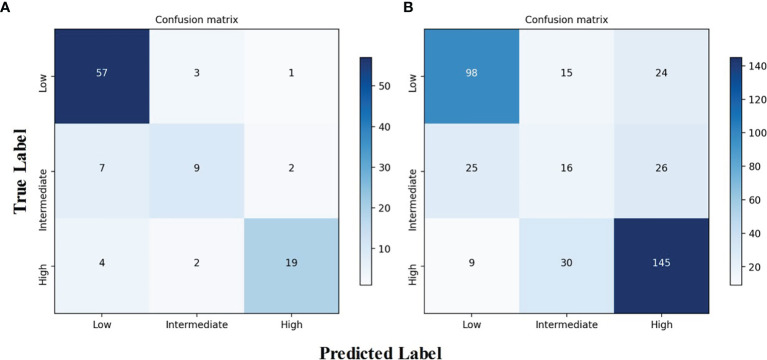
Confusion matrix of the DLM for risk stratification of gastrointestinal stromal tumors. **(A)** Confusion matrix for the testing cohort. **(B)** Confusion matrix for the independent external validation cohort. DLM, deep learning model.

The AUROCs for each grade were calculated to compare the model’s performance for each tumor risk stratification (**Table 2** and **Supplementary Results 2.1**). In the testing cohort, the ACC and AUROCs for each stratification was 86% (89 of 104, 95% CI: 80%, 92%) and 0.90 (95% CI: 0.84, 0.96) for low-malignant GISTs, 87% (90 of 104, 95% CI: 81%, 92%) and 0.80 (95% CI: 0.72, 0.88) for intermediate-malignant GISTs, and 91% (95 of 104, 95% CI: 86%, 97%) and 0.89 (95% CI: 0.83, 0.95) for high-malignant GISTs. In the external validation cohort, the ACC and AUROCs for each stratification was 81% (315 of 388, 95% CI: 77%, 85%) and 0.87 (95% CI: 0.83, 0.91) for low-malignant GISTs, 75% (292 of 388, 95% CI: 71%, 79%) and 0.64 (95% CI: 0.60, 0.68) for intermediate-malignant GISTs, and 77% (299 of 388, 95% CI: 73%, 81%) and 0.85 (95% CI: 0.81, 0.89) for high-malignant GISTs. The clinical validation results for the testing and external validation cohorts were summarized as confusion matrices for the GIST risk stratification predicted by the DLM compared with the pathologic risk stratification (**Figure 5**).

**Table 2:** Predictive performance of DLM in the testing and external validation cohorts.

Results	Accuracy (%)	Sensitivity (%)	Specificity (%)	F1 score (%)
Testing cohort				
Low-malignant	86 (89/104) [80, 92]	93 (57/61) [87, 99]	74 (32/43) [61, 88]	88
Intermediate-malignant	87 (90/104) [81, 92]	50 (9/18) [27, 74]	94 (81/86) [88, 100]	56
High-malignant	91 (95/104) [86, 97]	76 (19/25) [58, 94]	96 (76/79) [92, 100]	81
Overall result	82	73	88	75
External validation cohort				
Low-malignant	81 (315/388) [77, 85]	72 (98/137) [64, 79]	86 (217/251) [83, 90]	73
Intermediate-malignant	75 (292/388) [71, 79]	24 (16/67) [14, 34]	86 (276/321) [82, 90]	25
High-malignant	77 (299/388) [73, 81]	79 (145/184) [73, 85]	75 (154/204) [70, 81]	77
Overall result	67	58	83	58

Unless otherwise specified, data are percentages, with numbers of images in parentheses and 95% confidence intervals in brackets.

DLM, deep learning model.

Most stratification discrepancies occurred between the intermediate-malignant and non-intermediate-malignant GISTs. Due to the imbalanced number between the intermediate-malignant and non-intermediate GISTs (43 *vs.* 198) in the training dataset, the model training process may not fully be learned for the intermediate-malignant GISTs, which may lead to the DLM overfitting in the intermediate-malignant GISTs. Nevertheless, the DLM is able to discriminate the low-malignant GISTs and the high-malignant GISTs well. Taken together, the results achieve a great degree of agreement in the testing and external validation cohorts, which indicates the strong generalization capability of our proposed model.

A total of five loss functions were applied to optimize our grading model. The ablation experiments of loss function are shown in **Supplementary Results 2.2**.

### The Visualization of the Deep Learning Model

**Figure 6:**
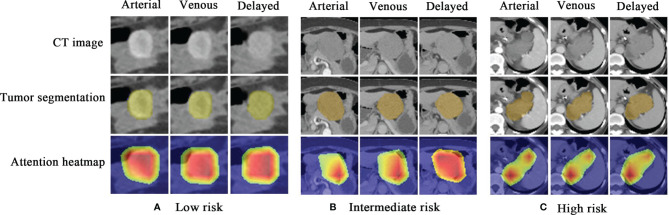
Attention heatmap drawn by gradient-weighted class activation mapping for the model interpretation. **(A)** CT images, tumor segmentations, and corresponding attention heatmaps in a 60-year-old woman with low-malignant GIST (the first column is arterial phase, the second column is venous phase, and the third column is delayed phase). **(B)** CT images, tumor segmentations, and corresponding attention heatmaps in a 66-year-old woman with intermediate-malignant GIST. **(C)** CT images, tumor segmentations, and corresponding attention heatmaps in a 43-year-old woman with high-malignant GIST. The red and yellow regions represent areas activated by the DLM and have the greatest predictive significance; the blue backgrounds reflect areas with weaker predictive values. GIST, gastrointestinal stromal tumor; DLM, deep learning model.

As shown in **Figure 6**, the attention heatmap highlights the relevant subregions for further clinical review, which indicates that the abnormal characteristics of the tumor have been learned by the DLM and used as the basis for its stratification of GIST risk categories.

### Comparison Between the Deep Learning Model and Radiomics Model

The DSC value of the arterial, venous, and delayed phases is 0.969, 0.973, and 0.967, respectively, which indicates that the two radiologists have a good agreement in the image segmentation.

One hundred sixty-one radiomics features were selected by LASSO, which were then enrolled to build the radiomics model. Thirty-seven features with feature importance ranking over 3 in five-fold are shown in **Supplementary Figure 3**. As shown in **Table 3**, the overall ACC of the testing and external validation cohorts is 75% (95% CI: 67%, 83%) and 68% (95% CI: 64%, 72%), respectively. The ROCs of the radiomics model used to evaluate the classification performance are shown in **Figures 4C, D**. In the testing cohort, the AUROCs for each stratification were 0.90 (95% CI: 0.84, 0.96) for low-malignant, 0.76 (95% CI: 0.68, 0.84) for intermediate-malignant, and 0.86 (95% CI: 0.80, 0.92) for high-malignant GISTs. In the external validation cohort, the AUROCs for each stratification were 0.89 (95% CI: 0.85, 0.93) for low-malignant, 0.57 (95% CI: 0.51, 0.63) for intermediate-malignant, and 0.87 (95% CI: 0.83, 0.91) for high-malignant GISTs.

**Table 3:** Predictive performance of radiomics model in the testing and external validation cohorts.

Results	Accuracy (%)	Sensitivity (%)	Specificity (%)	F1 score (%)
Testing cohort				
Low-malignant	83 (86/104) [75, 91]	98 (60/61) [94, 100]	60 (26/43) [47, 74]	87
Intermediate-malignant	84 (87/104) [76, 92]	6 (1/18) [2, 10]	100 (86/86) [100, 100]	11
High-malignant	84 (87/104) [76, 92]	68 (17/25) [50, 86]	89 (70/79) [81, 96]	67
Overall result	75	57	83	55
External validation cohort				
Low-malignant	80 (311/388) [76, 84]	87 (119/137) [81, 93]	77 (192/251) [71, 82]	76
Intermediate-malignant	80 (311/388) [76, 84]	9 (6/67) [3, 15]	95 (305/321) [93, 97]	14
High-malignant	76 (296/388) [72, 80]	76 (140/184) [70, 82]	77 (156/204) [71, 82]	75
Overall result	68	57	83	55

Unless otherwise specified, data are percentages, with numbers of images in parentheses and 95% confidence intervals in brackets.

Comparison of the performance of the DLM with the radiomics model revealed that the DLM displayed higher efficiency of diagnosis in the testing [AUROCs = 0.90 (95% CI, 0.84, 0.96) *vs.* 0.84 (95% CI, 0.76, 0.92)] and external validation (AUROCs = 0.81 (95% CI: 0.77, 0.85) *vs.* 0.78 (95% CI, 0.74, 0.81)) cohorts. Besides, in the testing cohort, the Obuchowski index value of DLM (Obuchowski index, 0.84) is significantly better than that of the radiomics model (Obuchowski index, 0.77) (p < 0.05), while no significant difference is found between the DLM (Obuchowski index, 0.79) and radiomics model (Obuchowski index, 0.77) in the external validation cohort.

## Discussion

The findings of our study show that the DLM could accurately predict the risk classification of GISTs with 0.90 AUROCs in the testing cohort. The performance in the external validation cohort was somewhat weaker but nevertheless very encouraging (AUROCs = 0.81). The performance of our proposed DLM is better than that of the radiomics model in both the testing and external validation cohorts, indicating that the DLM could mine more image features useful for assessing the risk classification in patients with GISTs. Our work represents an improved approach to the assessment of risk stratification based on the CT images from patients GISTs obtained before surgery and significantly improves on current prediction methods that rely on postoperative specimens.

To the best of our knowledge, this is the largest cohort study using deep learning for GIST risk stratifications and the only one distinguishing high-risk GISTs from intermediate-risk to low-risk GISTs. With few exceptions, reported model performance metrics in previous studies were focused on distinguishing low-malignant-potential GISTs (very low risk and low risk) from high-malignant-potential GISTs (intermediate risk and high risk), thus limiting their clinical impact for identifying high-risk GISTs (23). The European Society for Medical Oncology guidelines recommend adjuvant therapy for patients with a significant risk of relapse, with “room for shared decision-making when the risk is intermediate” (30). Joensuu et al. (1) reported that with modified NIH criteria, only high-risk patients might be considered for adjuvant treatment. Therefore, it is important to improve risk assessment in the high-risk GISTs to make more informed treatment decisions. Zhou et al. (10) indicated that the AUROCs of the multinomial logistic regression model for three risk degrees of GISTs (high-risk, intermediate-risk, and low-risk GISTs), established with three subjective CT features, was 0.806 (95% CI: 0.727, 0.885). In our study, the DLM demonstrated the AUROC value of 0.89 (95% CI: 0.83, 0.95) in the testing cohort and 0.85 (95% CI: 0.81, 0.89) in the external validation cohort for differentiating high-risk GISTs from intermediate-risk to low-risk GISTs, showing better performance than the subjective model.

Nevertheless, there exists a performance drop from the primary cohort (training and testing cohorts) to the external validation cohort, especially in the intermediate-malignant class as accuracy from 87% to 75%. Two main factors may account for the decreased performance: 1) for a three-class classification model, we adopted a one-*vs.*-rest method to evaluated the performance, which means that when the intermediate-malignant GISTs are masked as positive, the remaining two groups are regarded as negative. The sample size of intermediate-malignant GISTs is much less than that of the non-intermediate-malignant data, at around 1:5 in all datasets. This may hinder its performance, as the machine learning algorithms tend to be bias towards the majority class while exhibiting poor performance for the rest of the class. 2) There is a remarkable difference of CT scanner distribution between the acquisition of the primary and external validation cohorts, where future work could include a large variety of images from different CT scanners to further improve its generalizability. Deep learning (31) is a branch of artificial intelligence in which computers are not explicitly programmed but instead perform tasks by analyzing relationships between existing data points. More recently, deep learning algorithm-based image analysis has been applied to establish a direct link between diagnostic images and disease prediction (27, 32, 33). For example, Zhou et al. (34) recently demonstrated that a DLM based on ultrasound (US) images could provide an early diagnostic strategy for lymph node metastasis in patients with breast cancer. Choi et al. (35) showed that the deep learning system performed better than radiologists in the staging of liver fibrosis with CT images. In our study, we showed that a DLM with ResNet-based method was able to predict the risk classification of GISTs. Furthermore, the uninterpretable neural network system with applications in medical imaging is usually dubbed “black box” medicine (36). It is generally difficult to explain the internal relationship between input data and the predictive labels. The method of visualization with CAM can solve this problem by showing the predictive parts of the image. In our study, the output of CAM attention roughly covering the tumor indicates that the model could exactly locate on the tumor and could make a reliable and interpretable decision in the predictive ability.

In early studies, radiomics features were used for risk stratification of GISTs (22, 37–41). Therefore, in addition to deep learning, we also performed diagnosis using radiomics model for comparison. In the current study, 161 radiomics features were selected to build a radiomics model for predicting the risk classification of GISTs, which achieved acceptable performance in the testing (AUROCs = 0.84, 95% CI: 0.76–0.92) and external validation (AUROCs = 0.78, 95% CI: 0.74–0.81) cohorts. Our accuracy was comparable with that of Zhang et al. (21), who reported that the generated radiomics model demonstrated favorable performance for the risk stratifications of GISTs with an AUROC value of 0.809 (95% CI: 0.777–0.841) in the validation cohort. However, the handcrafted radiomics features can only reflect simple features of relatively low order and may lack the specificity to assess the risk classification (42). Notably, the proposed DLM (AUROCs; testing, 0.90; external validation, 0.81) in our study outperformed the radiomics model for risk classification of GISTs.

Our study has several limitations. First, this is a retrospective study, and the data are not balanced for risk stratification. The performance of the DLM may have been better if we had trained the DLM with an ideal training set including a large amount of CT data that were balanced across the different risk stratifications. Second, the DLM is not a fully automated model, as it requires manual tumor segmentation on the CT images. Third, although we performed the clinical validation of the DLM by using relatively large datasets, the generalizability of this assessment tool needs to be evaluated further. Translating technical success to meaningful clinical impact is the next major challenge. Thorough evaluation and further improvement would be required to evaluate the clinical benefits of the DLM in predicting the risk stratification of patients with GISTs.

In conclusion, we developed a DLM for predicting risk stratification on CT images in patients with GISTs. With further validation in a larger population and model calibration, our DLM has great potential to serve as an important decision support tool in clinical applications.

## Data Availability Statement

The raw data supporting the conclusions of this article will be made available by the authors, without undue reservation.

## Ethics Statement

The studies involving human participants were reviewed and approved by the institutional review board of both Shandong Provincial Hospital and The Affiliated Hospital of Qingdao University. Written informed consent for participation was not required for this study in accordance with the national legislation and the institutional requirements.

## Author Contributions

BK, XSY, and XW contributed to the conception and design of the study. XSY, HW, SQ, XS, and XXY organized the database. BK, CS, and XW assessed the image feature. QZ, YW, and FS performed the statistical analysis. BK wrote the first draft of the manuscript. XSY, SY, and XW wrote sections of the manuscript. All authors contributed to the article and approved the submitted version.

## Funding

The present study was supported by a grant from the Taishan Scholars Project (XW), National Natural Science Foundation of China Grant (81871354 and 81571672), and Academic Promotion Programme of Shandong First Medical University (2019QL023).

## Conflict of Interest

Authors QZ, YW, and FS were employed by United Imaging Intelligence.

The remaining authors declare that the research was conducted in the absence of any commercial or financial relationships that could be construed as a potential conflict of interest.

## Publisher’s Note

All claims expressed in this article are solely those of the authors and do not necessarily represent those of their affiliated organizations, or those of the publisher, the editors and the reviewers. Any product that may be evaluated in this article, or claim that may be made by its manufacturer, is not guaranteed or endorsed by the publisher.

## References

[1] Joensuu H Hohenberger P Corless CL. Gastrointestinal Stromal Tumour. Lancet (2013) 382:973–83. doi: 10.1016/S0140-6736(13)60106-3 23623056

[2] Parab TM DeRogatis MJ Boaz AM Grasso SA Issack PS Duarte DA. Gastrointestinal Stromal Tumors: A Comprehensive Review. J Gastrointest Oncol (2019) 10:144–54. doi: 10.21037/jgo.2018.08.20 PMC6351301 30788170

[3] Chan KH Chan CW Chow WH Kwan WK Kong CK Mak KF. Gastrointestinal Stromal Tumors in a Cohort of Chinese Patients in Hong Kong. World J Gastroenterol (2006) 12:2223–8. doi: 10.3748/wjg.v12.i14.2223 PMC4087650 16610025

[4] Nilsson B Bumming P Meis-Kindblom JM Oden A Dortok A Gustavsson B. Gastrointestinal Stromal Tumors: The Incidence, Prevalence, Clinical Course, and Prognostication in the Preimatinib Mesylate Era–A Population-Based Study in Western Sweden. Cancer (2005) 103:821–9. doi: 10.1002/cncr.20862 15648083

[5] Joensuu H. Risk Stratification of Patients Diagnosed With Gastrointestinal Stromal Tumor. Hum Pathol (2008) 39:1411–9. doi: 10.1016/j.humpath.2008.06.025 18774375

[6] Li J Gong JF Wu AW Shen L. Post-Operative Imatinib in Patients With Intermediate or High Risk Gastrointestinal Stromal Tumor. Eur J Surg Oncol (2011) 37:319–24. doi: 10.1016/j.ejso.2011.01.005 21277730

[7] Lin JX Chen QF Zheng CH Li P Xie JW Wang JB. Is 3-Years Duration of Adjuvant Imatinib Mesylate Treatment Sufficient for Patients With High-Risk Gastrointestinal Stromal Tumor? A Study Based on Long-Term Follow-Up. J Cancer Res Clin Oncol (2017) 143:727–34. doi: 10.1007/s00432-016-2334-x PMC11819384 28083710

[8] Inoue A Ota S Sato S Nitta N Shimizu T Sonoda H. Comparison of Characteristic Computed Tomographic Findings of Gastrointestinal and Non-Gastrointestinal Stromal Tumors in the Small Intestine. Abdom Radiol (NY) (2019) 44:1237–45. doi: 10.1007/s00261-018-1865-9 30600381

[9] Vernuccio F Taibbi A Picone D LAG L Midiri M Lagalla R. Imaging of Gastrointestinal Stromal Tumors: From Diagnosis to Evaluation of Therapeutic Response. Anticancer Res (2016) 36:2639–48. 27272772

[10] Zhou C Duan X Zhang X Hu H Wang D Shen J. Predictive Features of CT for Risk Stratifications in Patients With Primary Gastrointestinal Stromal Tumour. Eur Radiol (2016) 26:3086–93. doi: 10.1007/s00330-015-4172-7 26699371

[11] Li H Ren G Cai R Chen J Wu X Zhao J. A Correlation Research of Ki67 Index, CT Features, and Risk Stratification in Gastrointestinal Stromal Tumor. Cancer Med (2018) 7:4467–74. doi: 10.1002/cam4.1737 PMC6144253 30123969

[12] O’Neill AC Shinagare AB Kurra V Tirumani SH Jagannathan JP Baheti AD. Assessment of Metastatic Risk of Gastric GIST Based on Treatment-Naive CT Features. Eur J Surg Oncol (2016) 42:1222–8. doi: 10.1016/j.ejso.2016.03.032 27178777

[13] Wang JK. Predictive Value and Modeling Analysis of MSCT Signs in Gastrointestinal Stromal Tumors (GISTs) to Pathological Risk Degree. Eur Rev Med Pharmacol Sci (2017) 21:999–1005. 28338197

[14] Cannella R La Grutta L Midiri M Bartolotta TV. New Advances in Radiomics of Gastrointestinal Stromal Tumors. World J Gastroenterol (2020) 26:4729–38. doi: 10.3748/wjg.v26.i32.4729 PMC7459199 32921953

[15] Maldonado FJ Sheedy SP Iyer VR Hansel SL Bruining DH McCollough CH. Reproducible Imaging Features of Biologically Aggressive Gastrointestinal Stromal Tumors of the Small Bowel. Abdom Radiol (NY) (2018) 43:1567–74. doi: 10.1007/s00261-017-1370-6 29110055

[16] Kang B Sun C Gu H Yang S Yuan X Ji C. T1 Stage Clear Cell Renal Cell Carcinoma: A CT-Based Radiomics Nomogram to Estimate the Risk of Recurrence and Metastasis. Front Oncol (2020) 10:579619. doi: 10.3389/fonc.2020.579619 33251142 PMC7672185

[17] Wang H Zhang J Bao S Liu J Hou F Huang Y. Preoperative MRI-Based Radiomic Machine-Learning Nomogram May Accurately Distinguish Between Benign and Malignant Soft-Tissue Lesions: A Two-Center Study. J Magn Reson Imaging (2020) 52:873–82. doi: 10.1002/jmri.27111 32112598

[18] Gillies RJ Kinahan PE Hricak H. Radiomics: Images Are More Than Pictures, They Are Data. Radiology (2016) 278:563–77. doi: 10.1148/radiol.2015151169 PMC4734157 26579733

[19] Vernuccio F Cannella R Comelli A Salvaggio G Lagalla R Midiri M. Radiomics and Artificial Intelligence: New Frontiers in Medicine. Recenti Prog Med (2020) 111:130–5. doi: 10.1701/3315.32853 32157259

[20] Wang C Li H Jiaerken Y Huang P Sun L Dong F. Building CT Radiomics-Based Models for Preoperatively Predicting Malignant Potential and Mitotic Count of Gastrointestinal Stromal Tumors. Transl Oncol (2019) 12:1229–36. doi: 10.1016/j.tranon.2019.06.005 PMC6614115 31280094

[21] Zhang L Kang L Li G Zhang X Ren J Shi Z. Computed Tomography-Based Radiomics Model for Discriminating the Risk Stratification of Gastrointestinal Stromal Tumors. Radiol Med (2020) 125:465–73. doi: 10.1007/s11547-020-01138-6 32048155

[22] Chen T Ning Z Xu L Feng X Han S Roth HR. Radiomics Nomogram for Predicting the Malignant Potential of Gastrointestinal Stromal Tumours Preoperatively. Eur Radiol (2019) 29:1074–82. doi: 10.1007/s00330-018-5629-2 30116959

[23] Feng C Lu F Shen Y Li A Yu H Tang H. Tumor Heterogeneity in Gastrointestinal Stromal Tumors of the Small Bowel: Volumetric CT Texture Analysis as a Potential Biomarker for Risk Stratification. Cancer Imaging (2018) 18:46. doi: 10.1186/s40644-018-0182-4 30518436 PMC6280355

[24] Chen Z Xu L Zhang C Huang C Wang M Feng Z. CT Radiomics Model for Discriminating the Risk Stratification of Gastrointestinal Stromal Tumors: A Multi-Class Classification and Multi-Center Study. Front Oncol (2021) 11:654114. doi: 10.3389/fonc.2021.654114 34168985 PMC8217748

[25] Berenguer R Pastor-Juan MDR Canales-Vazquez J Castro-Garcia M Villas MV Mansilla Legorburo F. Radiomics of CT Features May Be Nonreproducible and Redundant: Influence of CT Acquisition Parameters. Radiology (2018) 288:407–15. doi: 10.1148/radiol.2018172361 29688159

[26] Hosny A Parmar C Quackenbush J Schwartz LH Aerts H. Artificial Intelligence in Radiology. Nat Rev Cancer (2018) 18:500–10. doi: 10.1038/s41568-018-0016-5 PMC6268174 29777175

[27] Esteva A Kuprel B Novoa RA Ko J Swetter SM Blau HM. Dermatologist-Level Classification of Skin Cancer With Deep Neural Networks. Nature (2017) 542:115–8. doi: 10.1038/nature21056 PMC8382232 28117445

[28] Kaiming H Xiangyu Z Shaoqing R Jian S. Deep Residual Learning for Image Recognition. 2016 IEEE Conf on Comput Vis Pattern Recognit (CVPR) (2016) 770–8. doi: 10.1109/cvpr.2016.90

[29] van Griethuysen JJM Fedorov A Parmar C Hosny A Aucoin N Narayan V. Computational Radiomics System to Decode the Radiographic Phenotype. Cancer Res (2017) 77:e104–7. doi: 10.1158/0008-5472.CAN-17-0339 PMC5672828 29092951

[30] Casali PG Abecassis N Aro HT Bauer S Biagini R Bielack S. Gastrointestinal Stromal Tumours: ESMO-EURACAN Clinical Practice Guidelines for Diagnosis, Treatment and Follow-Up. Ann Oncol (2018) 29:iv267. doi: 10.1093/annonc/mdy320 30188977

[31] LeCun Y Bengio Y Hinton G. Deep Learning. Nature (2015) 521:436–44. doi: 10.1038/nature14539 26017442

[32] Lee JH Ha EJ Kim JH. Application of Deep Learning to the Diagnosis of Cervical Lymph Node Metastasis From Thyroid Cancer With CT. Eur Radiol (2019) 29:5452–7. doi: 10.1007/s00330-019-06098-8 30877461

[33] Zhao W Yang J Sun Y Li C Wu W Jin L. 3d Deep Learning From CT Scans Predicts Tumor Invasiveness of Subcentimeter Pulmonary Adenocarcinomas. Cancer Res (2018) 78:6881–9. doi: 10.1158/0008-5472.CAN-18-0696 30279243

[34] Zhou LQ Wu XL Huang SY Wu GG Ye HR Wei Q. Lymph Node Metastasis Prediction From Primary Breast Cancer US Images Using Deep Learning. Radiology (2020) 294:19–28. doi: 10.1148/radiol.2019190372 31746687

[35] Choi KJ Jang JK Lee SS Sung YS Shim WH Kim HS. Development and Validation of a Deep Learning System for Staging Liver Fibrosis by Using Contrast Agent-Enhanced CT Images in the Liver. Radiology (2018) 289:688–97. doi: 10.1148/radiol.2018180763 30179104

[36] Castelvecchi D. Can We Open the Black Box of AI? Nature (2016) 538:20–3. doi: 10.1038/538020a 27708329

[37] Ren C Wang S Zhang S. Development and Validation of a Nomogram Based on CT Images and 3D Texture Analysis for Preoperative Prediction of the Malignant Potential in Gastrointestinal Stromal Tumors. Cancer Imaging (2020) 20:5. doi: 10.1186/s40644-019-0284-7 31931874 PMC6958787

[38] Ning Z Luo J Li Y Han S Feng Q Xu Y. Pattern Classification for Gastrointestinal Stromal Tumors by Integration of Radiomics and Deep Convolutional Features. IEEE J BioMed Health Inform (2019) 23:1181–91. doi: 10.1109/JBHI.2018.2841992 29993591

[39] Choi IY Yeom SK Cha J Cha SH Lee SH Chung HH. Feasibility of Using Computed Tomography Texture Analysis Parameters as Imaging Biomarkers for Predicting Risk Grade of Gastrointestinal Stromal Tumors: Comparison With Visual Inspection. Abdom Radiol (NY) (2019) 44:2346–56. doi: 10.1007/s00261-019-01995-4 30923842

[40] Yan J Zhao X Han S Wang T Miao F. Evaluation of Clinical Plus Imaging Features and Multidetector Computed Tomography Texture Analysis in Preoperative Risk Grade Prediction of Small Bowel Gastrointestinal Stromal Tumors. J Comput Assist Tomogr (2018) 42:714–20. doi: 10.1097/RCT.0000000000000756 30015796

[41] Liu S Pan X Liu R Zheng H Chen L Guan W. Texture Analysis of CT Images in Predicting Malignancy Risk of Gastrointestinal Stromal Tumours. Clin Radiol (2018) 73:266–74. doi: 10.1016/j.crad.2017.09.003 28969853

[42] Feng B Chen X Chen Y Lu S Liu K Li K. Solitary Solid Pulmonary Nodules: A CT-Based Deep Learning Nomogram Helps Differentiate Tuberculosis Granulomas From Lung Adenocarcinomas. Eur Radiol (2020) 30:6497–507. doi: 10.1007/s00330-020-07024-z 32594210

