# Supplementary material for: Preoperative CT-Based Deep Learning Model for Predicting Risk Stratification in Patients With Gastrointestinal Stromal Tumors
Source: Front Oncol. 2021 Sep 17;11:750875. doi: 10.3389/fonc.2021.750875 (PMC8496403; doi:10.3389/fonc.2021.750875)
Supplement: Supplementary file 1 [file DataSheet_1.docx]

Supplementary Material

# Supplementary Information

## Inclusion and exclusion criteria of the patient

The inclusion criteria were as follows: 1) patients who underwent surgery for GISTs with curative intent; 2) information of postoperative pathologically confirmed GISTs risk category available; 3) acquisition of standard contrast-enhanced CT within 30 days before surgery. The exclusion criteria were as follows: 1) preoperative therapy (radiotherapy, chemotherapy or chemoradiotherapy); 2) no preoperative contrast-enhanced CT or poor-quality CT images.

## CT Image Acquisition, Retrieving Procedure

The cases in Shandong Provincial Hospital underwent contrast-enhanced CT using the multidetector row CT systems (Aquilion ONE, TOSHIBA; Discovery 750, GE Healthcare; Somatom Definition Flash, Siemens Healthcare; Ingenuity CT, Philips). The acquisition parameters are as follows: 120 kV tube voltage, 250-400 mA (using automatic tube current modulation technique) tube current, 0.5 s or 0.6 s gantry rotation time, 80×0.5 mm or 64 × 0.625 mm detector collimation, a matrix of 512×512, and a pitch of 1.388 or 0.984. Axial images were reconstructed with a 5 mm slice thickness. An 80-90 mL volume of iodinated contrast medium (Omnipaque 350, GE Healthcare, Shanghai, China) was injected into the antecubital vein by a power injector at a rate of 3.0 mL/s. Pre-enhanced CT was first acquired, followed by three postcontrast CT scans obtained in arterial phase (25-30 s), portal venous phase (60 s) and delayed phase (90-120 s).

The patients in The Affiliated Hospital of Qingdao University were examined using the multidetector row CT systems (Somatom Sensation 64, Siemens Healthcare; iCT 256, Philips; Discovery 750, GE Healthcare; Aquilion ONE, TOSHIBA) using the following parameters: 0.5 s gantry rotation time, 120 kV tube voltage, 200 mAs or 250-400 mA (using automatic tube current modulation technique) tube current, 64×0.6 mm or 64×0.625 mm detector collimation, a matrix of 512×512, and a pitch of 1 or 1.375. Axial images were reconstructed with a 5 mm slice thickness. An 80-90 mL volume of iodinated contrast medium (Iopromide, Ultravist 370; Bayer, Germany) was injected into the antecubital vein by a power injector at a rate of 2.5 mL/s. Pre-enhanced CT was first acquired, followed by three postcontrast CT scans obtained in arterial phase (30 s), portal venous phase (60 s) and delayed phase (90-120 s).

There is a remarkable difference of CT scanner distribution between the acquisition of main dataset and the external validation dataset, as shown in Supplementary Figure 1.

## Image preprocessing

Rotation, scaling and flipping were applied to each image with a probability of 0.5. The angle of rotation along the axis x or y was randomly sampled in the range of (−10°, 10°). Thus, the scaling factor was randomly sampled in the range of (0.75, 1.25). Flipping was implemented randomly along each axis.

For the samples whose tumor size are less than 50mm, the image was firstly resampled to a fixed spacing of 0.7×0.7×5 mm^3^ and then cropped with the tumor as the center and with the patch size of 64×64×16. For the other samples, the tumor bounding box was first cropped from the original image and then zoomed to the patch size of 64×64×16.

## Development of the deep learning model

### Deep learning network for extracting risk stratification related features

Network Architecture

We used classical 3D extended version of residual network as our backbone network. In our framework, 3D SE-Residual Network is composed of four residual blocks, which is consisted of convolution layers and skip connection at each resolution level. In our dataset, since the within-slice resolution is much higher than the between-slice resolution of the contrast-enhanced CT scans, the anisotropic convolution operation was adopted to the residual network backbone to overcome the difficulties caused by the anisotropic spatial resolution of medical images. In our study, the normal isotropic convolutions with kernel size of 3×3×3 were replaced by a combination of 1×3×3 and 3×1×1 continuous anisotropic convolutions in the first three residual blocks. The fourth residual block still used the normal isotropic convolutions with kernel size of 3×3×3, since the gap between within-slice and between-slice resolution of the output feature maps of the third anisotropic residual block is negligible.

Attention module

In our framework, we adopted two attention modules to guide the network to focus on the tumor region and to learn grading related discriminative features. First, the well-known class activation mapping (CAM) was used as an online attention mechanism, where the attention maps are generated by back-propagating weights of the fully-connected layer onto the convolutional feature maps[1]. Second, each residual block is embedded with a squeeze-and-excitation network (SE-Net), which is designed to improve the representational power and the discriminative performance of the SE-Residual Network[2].

Loss functions

In this stage, the function of SE-Residual Network is to make the discriminative decision of each tumor sample for all sequences coming from in the training process. The cross-entropy loss $L_{ce}$ was adopted for tumor grading task. To guide the network focuses on the tumor region, an extra attention loss $L_{att}$ was defined in the form of dice loss between the attention map and the tumor mask. Considering the class-imbalance problem, focal loss $L_{fl}$[[3](#_ENREF_3)] and batch nuclear-norm maximization (BNM) loss $L_{bnm}$ were introduced to improve both discriminability and diversity of the model decision during optimization. Moreover, the center loss $L_{cl}$ was also introduced to penalize the distances of the intra-class features since the multi-sequence deep features of the same tumor should be close, and keep the invariance of the rotation, transformation and scaling.

Given a batch of input images $I=[I_{1},\cdots, I_{b}, \cdots,I_{B}]$ and the corresponding tumor masks $M=[M_{1},\cdots, M_{b}, \cdots,M_{B}]$, where $B$ is the batch size. Denote $Y=[y_{1},\cdots, y_{b}, \cdots,y_{B}]$ as the corresponding labels and $P=[p_{1},\cdots, p_{b}, \cdots,p_{B}]$ as the corresponding predicted feature maps, where $p_{b}=[p_{b1},\cdots, p_{bc}, \cdots,p_{BC}]$, $C$ is the number of class, denote $T=[T_{1},\cdots, T_{b}, \cdots,T_{B}]$ as the corresponding attention maps and $F=[f_{1},\cdots, f_{b}, \cdots,f_{B}]$ as the corresponding deep features, denote $V=[v_{1},\cdots, v_{c}, \cdots,v_{C}]$ as the class center vector of deep features, which are be learned in the training process. The overall loss function $L_{total}$ is formulated as follows:

| $L_{total}=L_{ce}+\alpha L_{att}+\beta L_{fl}+\mu L_{bnm}+\delta L_{cl}$ | (1) |
| --- | --- |
| $L_{ce}=-\frac{1}{B}\sum_{b} y_{b}log(p_{b})$ | (2) |
| $L_{att}=\frac{1}{B}(1-\sum_{b} \frac{2\sum_{ijk} \left( M_{bijk}T_{bijk} \right)}{\sum_{ijk} M_{bijk}+\sum_{ijk} T_{bijk}})$ | (3) |
| $L_{fl}=-\frac{1}{B}\sum_{b} {(1-p_{b}y_{b})}^{\gamma}log(p_{b})$ | (4) |
| $L_{bnm}=-\frac{1}{B}\left\Vert P \right\Vert_{*}$ | (5) |
| $L_{cl}=\frac{1}{2B}\sum_{b} \sum_{c} \left\Vert F_{y_{b}=c}-v_{c} \right\Vert_{2}^{2}$ | (6) |

where $\left\| P \right\|_{*}$ represents the value of nuclear-norm, $i,j,k$ represent the ${(i,j,k)}^{th}$ voxel in the tumor masks and the attention maps. In our study, $\alpha,\beta,\mu,\delta,\gamma$ are set as 0.5, 1, 1, 0.001, and 2, respectively.

### The decision network for patient diagnosis

Network Architecture

In the proposed framework, the classification network is consisted of two fully connected layers, where the dimension of the input feature vectors is firstly reduced to 256 by the first fully connected layer, and then reduce to the number of class by the second fully connected layer.

Loss function

In this stage, the function of the classification network is to make the final tumor diagnosis for each subject in the training process. The cross-entropy loss $L_{ce}$ described in formula (2) is adopted for patient diagnostic task.

### Training details

The Adam optimizer was applied to optimize the model with a momentum set to 0.9, a weight decay of 0.0001, and a learning rate of 0.001 that is reduced by a factor of 10 after every 50 epochs. We set the batch size as 3 during the training.

## The details of the radiomics features

The radiomics features are obtained through a python package named Pyradiomics (https://pyradiomics.readthedocs.io). There are 104 features that could be extracted from a given image, including 7 categories, i.e., first-order image intensity statistics, shape, gray level co-occurrence matrix (GLCM), gray-level run-length matrix (GLRLM), gray-level size-zone matrix (GLSZM), neighboring gray tone difference matrix (NGTDM), and gray-level dependence matric (GLDM). In this work, we employed 25 image filters, i.e., original image, additive Gaussian noise, box mean, binomial blur, box sigma, curvature flow, normalize, Laplacian sharpening, discrete Gaussian, mean, speckle noise, recursive Gaussian, shot noise, wavelet (8 filters) and Laplacian of Gaussian (4 filters). In this way, from a given image, a total of 2600 (104*25) features are extracted.

# Supplementary results

## Results of the separated risk stratification in the testing and external validation cohorts

To compare the model’s performance for each tumor risk stratification in the testing cohort and external validation cohort, the results of the separated risk stratification are shown in Table 3. To be specific, for examples, the evaluated results of the low-malignant GISTs is calculated based on the binary classification through dividing the dataset into the low-malignant and the non-low-malignant GISTs. The results of the intermediate-malignant GISTs and the high-malignant GISTs is also calculated by means of the same binary classification as the low-malignant GISTs. As shown in Table 3, for the low-malignant GISTs in the external validation cohort, the results of AUROC, accuracy are comparable to those in the testing cohort, and the results of sensitivity and F1-score have a decrease by larger than 15%, whereas the result of specificity gains an increase by larger than 10%, compared to those in the testing cohort. For the intermediate-malignant GISTs in the external validation cohort, the results of AUROC, accuracy, sensitivity, specificity, F1-score are all decreased greatly compared to those in the testing cohort. For the high-malignant GISTs in the external validation cohort, the results of AUC and F1-score are comparable to those in the testing cohort, and the results of accuracy and specificity exhibit a great decrease, whereas the result of sensitivity gain an increase by 3%, compared to those in the testing cohort.

## Ablation experiments of loss function

We first investigated the impact of different combination of loss functions in the proposed framework. In this study, a total of five loss functions are applied to optimize our grading model. To further investigate the effect of $L_{bnm}$ and $L_{cl}$, an ablation experiments of the two loss functions are deployed, and evaluated in the testing cohort (Supplementary Table 2). The contribution of $L_{bnm}$ is reflected in the improvement of accuracy and specificity. Moreover, the function of the single $L_{cl}$ and the combination of $L_{bnm}$ and $L_{cl}$ is shown the opposite effect. The ROC curves of models optimized by four different combination of loss functions in the testing cohort are illustrated in Supplementary Figure 4.

# Supplementary Figures and Tables

## Supplementary Figures

**Supplementary Figure 1.** The difference of CT scanner distribution between the acquisition of main dataset and the external validation dataset


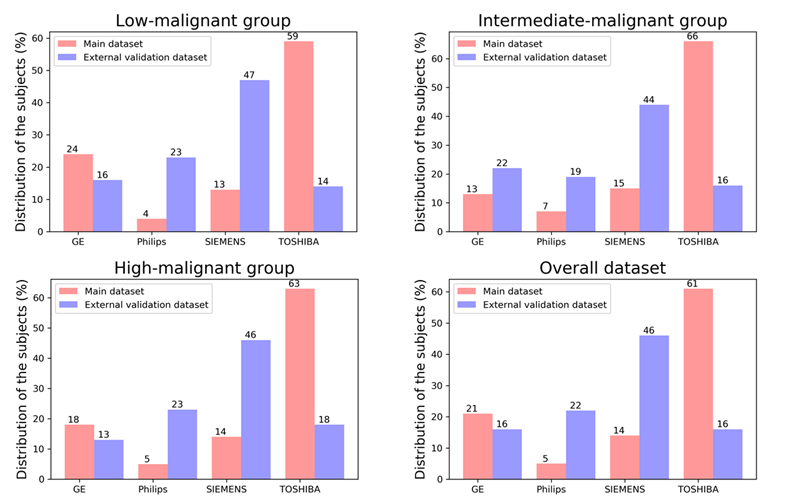


**Supplementary Figure 2.** The histogram of the tumor size distribution in the dataset of primary and external validation cohorts.


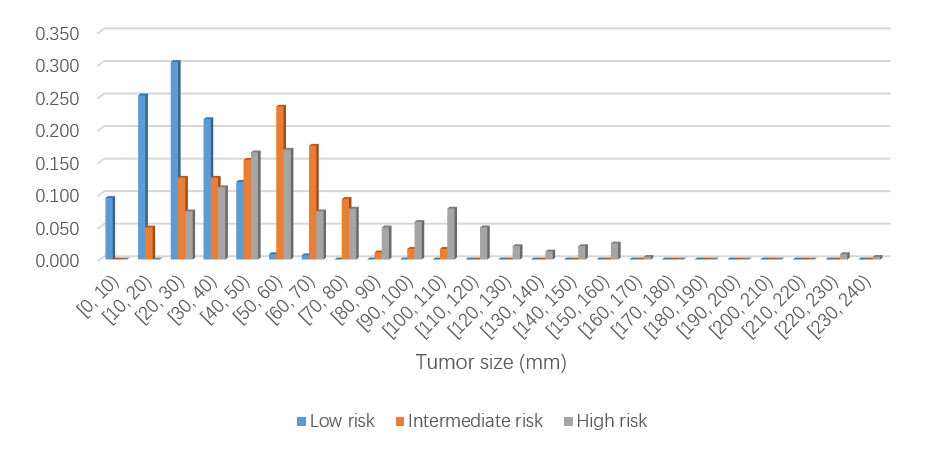


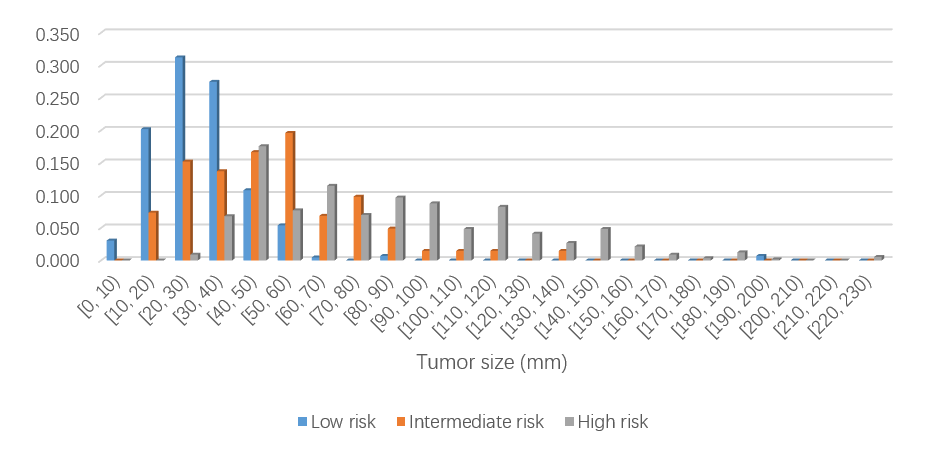


**Supplementary Figure 3.** Features importance ranking of Radiomics features over 3 by LASSO in 5 folds (The last character of each feature A represents arterial phase, D represents delayed phase, and V represents venous phase).

**
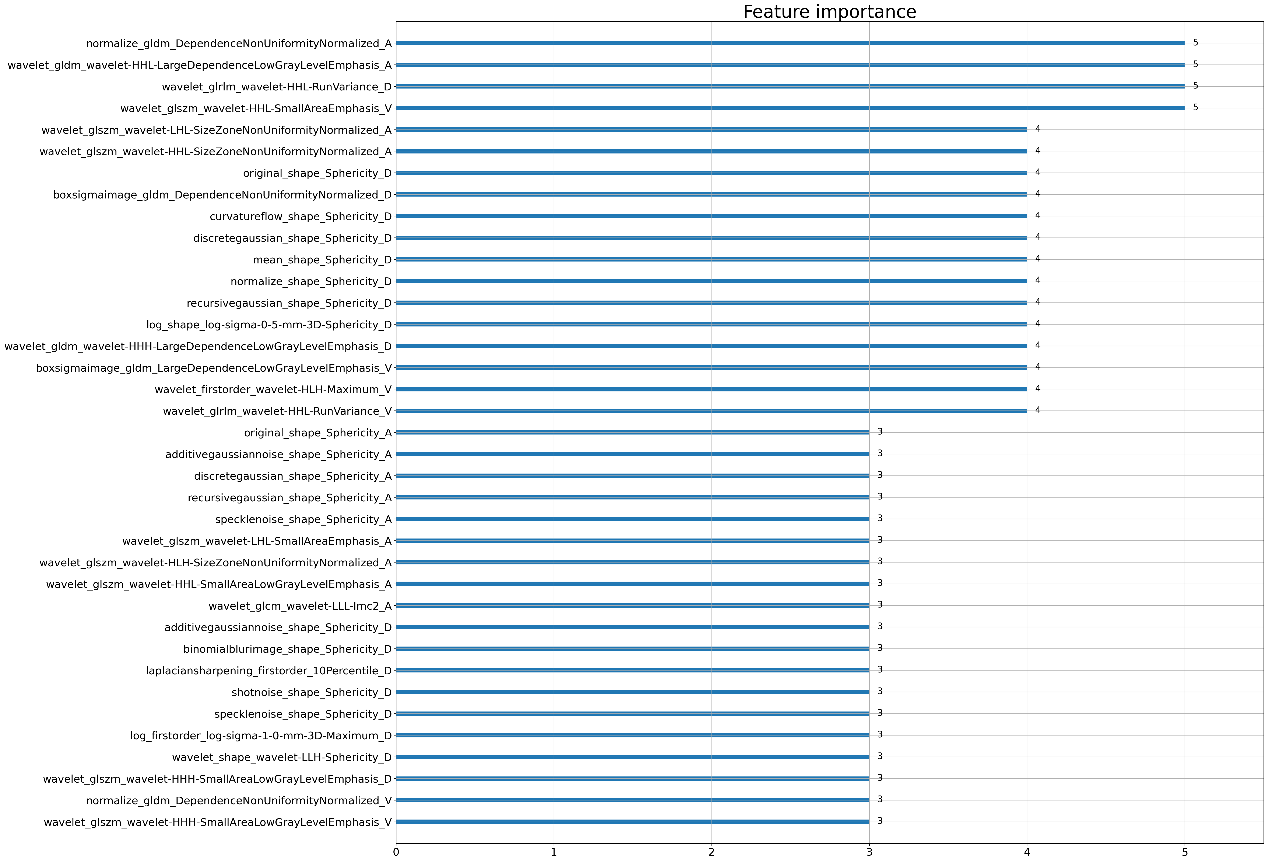
**

**Supplementary Figure 4.** The ROC curves of models optimized by different combination of loss functions in the testing cohort.


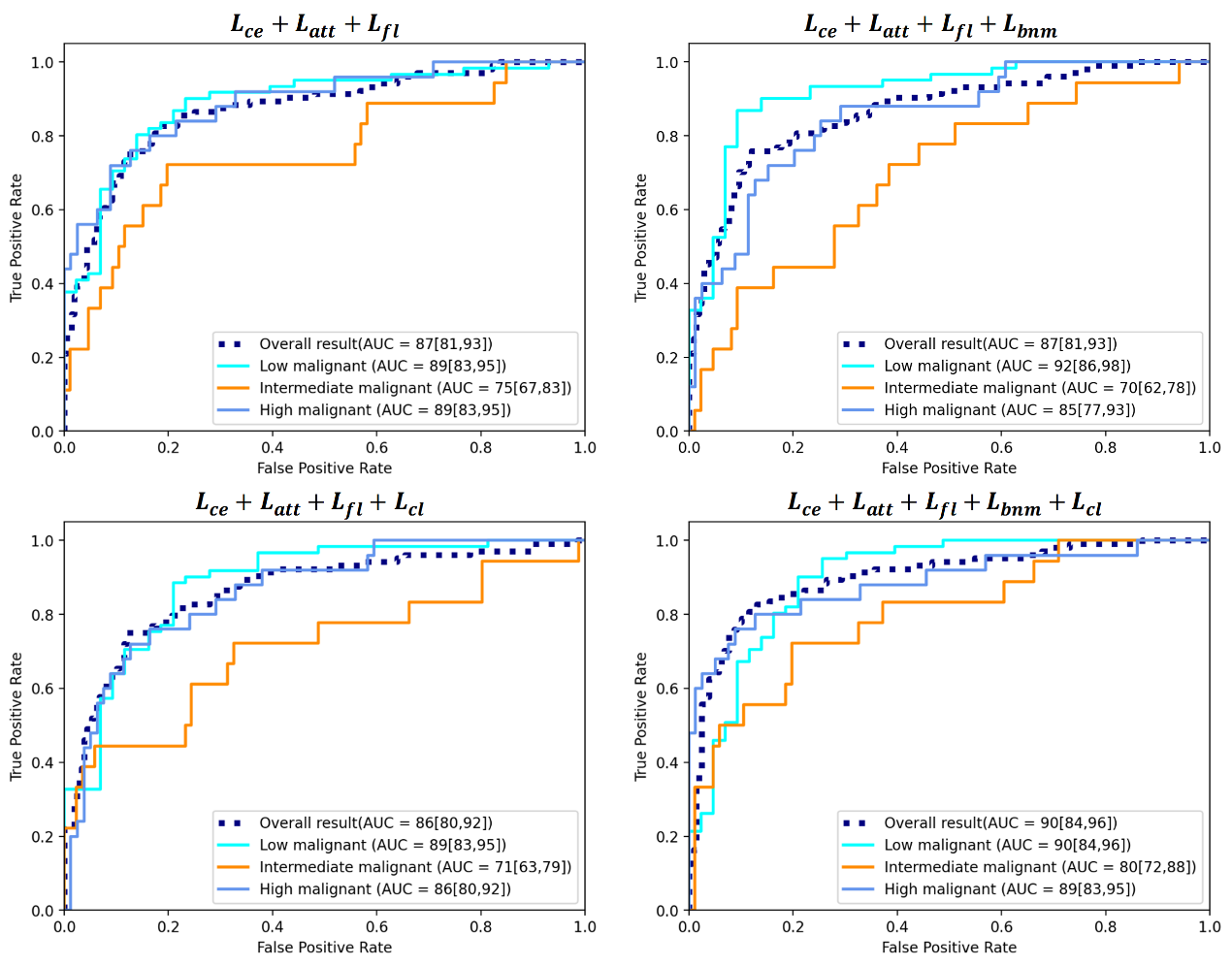


## Supplementary Table

**Supplementary Table 1.** Modified NIH risk of recurrence

| Risk | Tumor size (cm) | Mitotic count (HPF) | Tumor site |
| --- | --- | --- | --- |
| Very low risk | <2 | <5/50 | Any |
| Low risk | 2.1-5.0 | <5/50 | Any |
| Intermediate risk | <5 | 6-10/50 | Gastric |
|  | 5.1-10.0 | <5/50 | Gastric |
| High risk | Any | Any | Tumor rupture |
|  | >5 | >5/50 | Any |
|  | >10 | Any | Any |
|  | Any | >10/50 | Any |
|  | 2.1-5.0 | >5/50 | Non-gastric |
|  | 5.1-10.0 | <5/50 | Non-gastric |

HPF, high power field

**Supplementary Table 2.** Ablation results of models optimized by different combination of loss functions in the testing cohort.

| **Results** | **AUC** | **ACC** | **SEN** | **SPE** | **F1** |
| --- | --- | --- | --- | --- | --- |
| $L_{ce}+L_{att}+L_{fl}$ | 0.87 | 0.75 | 0.68 | 0.86 | 0.69 |
| $L_{ce}+L_{att}+L_{fl}+L_{bnm}$ | 0.87 | 0.77 | 0.68 | 0.88 | 0.67 |
| $L_{ce}+L_{att}+L_{fl}+L_{cl}$ | 0.86 | 0.75 | 0.66 | 0.86 | 0.66 |
| $L_{ce}+L_{att}+L_{fl}+L_{bnm}+L_{cl}$ | 0.90 | 0.82 | 0.73 | 0.88 | 0.75 |

**Reference**

1. Ouyang X, Huo J, Xia L, Shan F, Liu J, Mo Z, et al. Dual-Sampling Attention Network for Diagnosis of COVID-19 From Community Acquired Pneumonia. *IEEE Trans Med Imaging* 2020;39:2595-2605

2. Hu J, Shen L, Albanie S, Sun G, Wu E. Squeeze-and-Excitation Networks. *IEEE Trans Pattern Anal Mach Intell* 2020;42:2011-2023

3. Lin TY, Goyal P, Girshick R, He K, Dollar P. Focal Loss for Dense Object Detection. *IEEE Trans Pattern Anal Mach Intell* 2020;42:318-327
